# Persistence of pain and suffering in cancer patients: challenges of pain management from the perspective of nurses

**DOI:** 10.3389/fpain.2024.1425036

**Published:** 2024-09-05

**Authors:** Parvin Mangolian Shahrbabaki, Jamileh Farokhzadian, Fazlollah Ahmadi, Fatemeh Khabbazzadeh

**Affiliations:** ^1^ Nursing Research Center, Kerman University of Medical Sciences, Kerman, Iran; ^2^ Nursing Department, Faculty of Medical Sciences, Tarbiat Modares University, Tehran, Iran; ^3^ Reproductive Health, Family and Population Research Center, Kerman University of Medical Sciences, Kerman, Iran

**Keywords:** challenges, cancer, pain management, qualitative study, nurses

## Abstract

**Purpose:**

Cancer patients often experience pain, which can greatly reduce their quality of life. It affects their emotions, cognitive function, and daily interactions. Healthcare providers need to understand the obstacles to pain management to create helpful programs for patients and families. This study focuses on Iranian nurses' views on pain management barriers in cancer patients.

**Methods:**

The study utilized a qualitative content analysis method with a purposive sampling approach, involving 27 nurses. Participants were selected to ensure a wide range of perspectives and experiences by considering factors such as gender, age, work experience, education levels, and positions until data saturation was achieved. Inclusion criteria specified a minimum of six months of oncology ward experience and a bachelor's degree or higher in nursing. Nurses with less than six months of oncology experience or lacking a nursing degree were excluded. Data collection was conducted through semi-structured interviews and analyzed using Lundman and Granheim's qualitative content analysis method.

**Results:**

One main theme, four main categories, and nine subcategories in the study reflected the nurses' experiences and viewpoints about barriers to pain management. These categories included the marginalization of complementary medicine, medical malpractice, inadequate organizational infrastructure, and personal barriers.

**Conclusion:**

The study demonstrated that the barriers to pain management in cancer patients were complex. To improve patients' comfort and quality of life, it is important to identify and address issues from different angles. It is crucial to train patients and healthcare providers in pain management and to address weaknesses in the healthcare system.

## Introduction

Pain is prevalent among cancer patients, particularly those in advanced stages, affecting all aspects of life (1). It reduces quality of life by impacting emotions, cognitive function, daily functioning, and communication (2). Approximately 70% of cancer patients experience severe pain, depending on the type and stage of cancer (3). While cancer may be perceived as a terminal illness, patients deserve a pain-free life, and efforts should be made to alleviate their suffering (4). Effective pain management can significantly improve comfort and functionality for cancer patients (5).

Pain management involves reducing or controlling pain through assessment, treatment, education, and psychological support (6). In advanced stages of cancer, managing pain becomes crucial, especially towards the end of life (7). Effective pain management can help approximately 90% of cancer patients (6). Despite available medications and guidelines, many patients are dissatisfied with pain control (8). Studies show that the pain of cancer patients is poorly managed (9).

Quantitative studies have identified various barriers to pain management for cancer patients, including insufficient knowledge (10), attitudes (11), staffing issues (12), cultural differences (13), and the absence of a clear clinical model (14). Time constraints, knowledge gaps, and inadequate nursing care also pose challenges for pain management in cancer patients (15, 16).

In Iran, cancer is a prevalent disease, with projections indicating that it will account for 13.4% of deaths in the country by 2030. Annually, Iran reports 80,000 new cancer cases and 30,000 cancer-related deaths, underscoring the increasing need for specialized care (17). Cancer patients in Iran experience various emotional, psychological, and physical symptoms, including pain, impacting their quality of life (14). Studies on nurses' pain management in Iran are limited, with healthcare facilities mainly relying on medical models instead of specific nursing models for effective pain management.

Quantitative studies may not fully capture the complexities of pain management due to its qualitative nature. Therefore, employing qualitative research can provide a more comprehensive understanding of pain management through nurses' experiences. Qualitative research offers rich and detailed insights into interactive processes, behavioral causes, and development of practical theories. Given nurses' limited knowledge and the influence of racial, cultural, and ethnic factors, it is essential to explore pain management within the natural context and structure, taking into account influencing factors (18).

Complex process of pain management for cancer patients is influenced by human and environmental factors, interactions among the patient, family, and healthcare team, and social interactions. This aligns with the theory of symbolic interactionism (19). Therefore, a qualitative research method was employed to elucidate the obstacles to pain management for Iranian cancer patients from the perspective of nurses, with the aim of gaining a comprehensive understanding of this critical issue.

### Review of literature

We performed a comprehensive analysis of current studies on cancer pain management. The findings showed a significant gap in this field. Existing theories fall short in addressing the individual context of cancer pain and lack specific strategies to enhance pain management for cancer patients. When treating cancer patients, it is crucial to identify pain during the management process and utilize models or theories that provide this support. Healthcare professionals should consider these therapeutic models in the social and cultural context, ensuring pain management aligns with Iranian culture to establish a suitable environment.

## Study design

The current qualitative study, conducted in 2023, aimed to explore the barriers to pain management in Iranian cancer patients from the perspective of nurses. This qualitative approach was chosen to delve into the nurses’ experiences and address the main questions, allowing for a comprehensive exploration of the topic.

### Participants

The focus in qualitative research is on identifying rich sources for study participation. As the study progresses, new questions arise, such as determining who else should be engaged with to enhance understanding of the subject. Therefore, purposive sampling was utilized for this study (20). Nurses working in oncology departments of hospitals affiliated with the Yazd University of Medical Sciences were purposefully selected as participants. A total of 27 nurses meeting specific criteria took part in the study. The first researcher, along with nurse managers, identified eligible participants. The sampling process continued based on the principle of maximum variation to capture rich and diverse perspectives and experiences of the participants. Therefore, nurses with different genders, ages, years of work experience, degrees, and positions were enrolled until data saturation. Inclusion criteria required the nurses to have a minimum of six months of work experience in oncology wards and hold a bachelor's degree or higher in nursing. Nurses who had less than six months of work experience in the oncology department or did not have a university degree in nursing were excluded from the study. Sampling continued until data saturation was reached, with no dropouts.

### Data collection

In this study, data were collected from July to October 2023. Data collection was done through open semi-structured interviews. The first researcher, a doctoral nursing student, with clinical experience and qualitative study familiarity, conducted all interviews. They approached nurses in various departments, explained the study's goals, invited participation, and scheduled interview sessions. Face-to-face interviews were audio recorded, transcribed, and coded using Max Q software. Thematic guiding questions were developed collaboratively by the research team to align with the study objectives. Starting with engaging questions to create a comfortable environment, the interviews explored nurses' experiences and perspectives on managing cancer patients’ pain and effective factors of pain management. Some guiding questions included descriptions of pain management experiences, treatment approaches for cancer pain, and factors contributing to successful pain management. Follow-up questions were used to delve into participants’ responses and gather deeper insights, such as “Please tell me more about…” Interviews were conducted with nurses, respecting their schedules and preferences for interview locations within hospitals. Each interview lasted about 40–65 min in a quiet room near the ward or another location in the hospitals.

### Setting

The hospitals affiliated with Yazd University of Medical Sciences serve as vital centers for cancer treatment in southern and southeastern Iran. Patients from neighboring areas often seek treatment there, exposing nurses to diverse cultures and lifestyles. These cultural variations can impact the care provided by nurses, prompting the decision to focus on researching nurses in these hospitals.

### Approach

The data analysis in this study was conducted using the method proposed by Graneheim and Lundman, which aims to achieve a comprehensive and thorough understanding of the phenomenon through qualitative content analysis (21).

### Data analysis

The interview text served as the primary data for the research, initially transcribed verbatim. Subsequently, the text was broken down into meaning units, which were then summarized and condensed. Next, the meaning units were abstracted and the codes were selected based on both explicit and implicit concepts derived from participants' statements. Constant comparison was used to group codes representing similar topics into distinct categories. Ambiguous points were also examined in subsequent interviews. At an interpretive level, summary categories and central concepts of each category were defined, and key abstract concepts were extracted. The concepts were based on internal themes described across all data (22). Table 1 shows an example of the analysis process used in this study. Throughout the data collection and analysis, the researcher recorded any reflections or remarks related to the data for future reference.

**Table 1:** An example of qualitative content analysis.

Category	Subcategories	Codes	Meaning units
Medical malpractice	Inattention given to pain management process	Failing to examine a patient with abdominal pain can exacerbate the patient's condition.	In some cancer patients, abdominal pain caused by liver damage may lead nurses to administer analgesics without promptly assessing the abdomen or determining the root cause of the pain. This approach could exacerbate the patient's liver condition.
	Disadvantages of doctor visits	The patient's pain continues due to the delay in visiting	Sometimes, interns or residents may be absent from the hospital, especially during night shifts, resulting in extended delays in patient reassessment, causing patients to endure prolonged pain.
		Limiting the prescription of analgesics below the patient's actual requirement prevents effective pain management for the patient.	Although doctors usually prescribe analgesics based on standard formulas and medical literature guidelines, patients with similar conditions may have different physiological requirements, necessitating varied analgesic doses. Inadequate painkiller dosages can hinder effective pain management for these patients.

The study's trustworthiness was assessed using the criteria outlined by Guba and Lincoln (22). Participants and supervisors ensured data accuracy and reliability through code reviews. The researcher was deeply engaged with the subject, data, and nurses for over a year. Before the interviews, the researcher visited each participant to build trust and prepare for in-depth interviews. Participants were provided with a portion of the text and initial coding for comparison to verify consistency between researcher interpretations and their original statements. Peer checking was conducted by presenting developed concepts and categories.

## Results

### Demographic information

The participants included two head nurses, 24 clinical nurses, and a clinical supervisor. The nurses were 19 women and eight men aged 25–54 years with work experiences ranging from 1 to 30 years. Three nurses had a master's degree and the others had a bachelor's degree in nursing (Table 2).

**Table 2:** Demographic characteristics of the participants in the study.

Participant	Gender	Age-ranges	Position	Work experience-ranges	Degree	Participant	Gender	Age-ranges	Position	Work experience-ranges	Degree
1	F	37–46	Head nurse	21–30	Master	15	F	33–42	Clinical nurse	11–20	Bachelor
2	F	33–42	Clinical nurse	11–20	Bachelor	16	F	25–34	Clinical nurse	11–20	Bachelor
3	F	25–34	Clinical nurse	1–10	Bachelor	17	F	25–34	Clinical nurse	1–10	Bachelor
4	M	37–46	Clinical nurse	21–30	Bachelor	18	M	33–42	Clinical nurse	11–20	Bachelor
5	F	37–46	Clinical nurse	11–20	Master	19	F	33–42	Clinical nurse	11–20	Bachelor
6	M	33–42	Clinical nurse	11–20	Bachelor	20	F	37–46	Clinical nurse	21–30	Master
7	M	25–34	Clinical nurse	1–10	Bachelor	21	F	33–42	Clinical nurse	11–20	Bachelor
8	F	33–42	Clinical nurse	11–20	Bachelor	22	F	33–42	Clinical nurse	11–20	Bachelor
9	F	37–46	Clinical nurse	21–30	Bachelor	23	M	25–34	Clinical nurse	1–10	Bachelor
10	F	33–42	Clinical nurse	11–20	Bachelor	24	F	25–34	Clinical nurse	1–10	Bachelor
11	F	25–34	Clinical nurse	1–10	Bachelor	25	F	37–46	Clinical nurse	1–10	Bachelor
12	F	25–34	Clinical nurse	1–10	Bachelor	26	M	33–42	Clinical nurse	11–20	Bachelor
13	M	46–54	Head nurse	21–30	Bachelor	27	F	33–42	Clinical nurse	21–30	Bachelor
14	M	46–54	Clinical supervisor	21–30	Master						

### Results from interviews

One main theme, four main categories, and nine subcategories in the study reflected the nurses' experiences and viewpoints about barriers to pain management. These categories included the marginalization of complementary medicine, medical malpractice, inadequate organizational infrastructure, and personal barriers (Table 3).

**Table 3:** Barriers to pain management in cancer patients from the perspective of nurses.

Main them	Main category	Subcategory
Barriers to pain management in cancer patients	Marginalization of complementary medicine	Disbelief in complementary medicine for pain relief
		Lack of knowledge and skills in complementary medicine
	Medical malpractice	Lack of attention to pain management process
		Problems related to medical visits.
	Inadequate organizational infrastructure	Lack of personnel
		Inappropriate environment
	Personal barriers	Treatment resistance
		Fear of drug side effects
		Patients’ lack of knowledge

Results along with quotations presented by participants are described in the following.

### Marginalization of complementary medicine

A key factor leading to the underutilization of pain management methods was the segregation of complementary medicine among healthcare providers. This study exposed healthcare professionals' skepticism towards the palliative benefits of complementary medicine and the insufficient knowledge and skills among providers regarding non-pharmacological pain management methods, contributing to the marginalization of complementary medicine in alleviating patients' pain.

#### Healthcare professionals’ skepticism towards the palliative benefits of complementary medicine

The use of complementary medicine for pain relief requires Physicians' cooperation and acceptance of this practice. Data analysis revealed that physicians often do not believe in the efficacy of complementary medicine methods for relieving pain in cancer patients. They argue that the pain intensity of these patients requires strong painkillers or sedation methods. This perspective serves as an obstacle to specialists' adoption and recommendation of complementary medicine practices.

*“Complementary medicine methods are not used at all in the hospital because most doctors have not yet recognized complementary medicine.” (P6)*

#### Insufficient knowledge and skills in complementary medicine

Effective use of complementary medicine methods for pain management requires having sufficient knowledge about these methods and their possible effects, as well as having the necessary skills for their application. In this study, the majority of participants observed a notable deficiency among healthcare workers in terms of interest, knowledge, and comprehension of pain relief, alongside the skills needed for employing non-pharmacological methods.

*“I have never seen non-pharmacological methods used to relieve patients’ pain. The personnel are not skilled in non-pharmacological methods and immediately resort to prescribing painkillers.” (P10)*

### Medical malpractice

Effective pain management requires a team approach, involving physicians and nurses in diagnosing, assessing, and implementing treatment for patients' pain. Physicians prescribe painkillers, while nurses assess pain levels and determine the appropriate use of painkillers and non-pharmacological methods. Participants highlighted that lack of attention to the pain management process and instances of medical malpractice among healthcare providers were substantial barriers to effective pain management.

#### Nurses' lack of attention to the pain management process

Effective pain management is essential for alleviating patients’ suffering and improving their quality of life. However, the experiences of nurses show that a lack of communication or inappropriate behavior from physicians may prevent nurses from reporting pain accurately. Challenges in filling out pain assessment forms further impede proper pain management. Participant experiences highlighted instances where some nurses displayed indifference towards pain management, occasionally administering analgesics without doctor orders. Such actions could lead to severe patient harm or delays in pain management processes.

*“Sometimes, nurses administer analgesics to alleviate a patient’s pain without proper examination or order, simply to provide relief. Unfortunately, this practice has, at times, worsened the patient's condition and introduced new problems.” (P4)*

#### Disadvantages of doctor visits

Effective pain management requires physicians to accurately and promptly assess patients in pain and prescribe analgesics based on a correct diagnosis of their pain needs, including the type and dosage of analgesics. The experiences of the nurses in this study revealed that some doctors were indifferent to monitoring patients' pain or caused delays in attending to them, preventing effective pain management. Other barriers included inadequate examination and evaluation by physicians, as well as lack of attention to prescribing the appropriate dose of analgesics due to physiological differences.

*“Specialists or residents’ visits are very brief. The dosage of analgesics does not seem to be based on accurate and thorough examination or individual differences.” (P 5*(

“*Sometimes, doctors’ visits may be superficial as they perceive pain as inherent to the disease, considering it normal for patients to experience pain without emphasizing its significance.” (P8)*

### Inappropriate organizational infrastructures

Organizational infrastructures significantly influence the pain management of patients. Factors such as an adequate number of nursing staff, sufficient analgesics, and a suitable environment for patient rest after receiving painkillers play crucial roles in the pain relief process. Lack of proper rest post-medication can compromise the effectiveness of painkillers. Additionally, insufficient nursing staff may lead to the neglect or delayed care for patients in pain due to competing responsibilities. Nurses in this study highlighted inappropriate organizational infrastructure, lack of personnel, and inadequate environments as key subcategories affecting the pain management of cancer patients.

#### Lack of personnel

Insufficient personnel in the department lead to an unstructured division of labor and inappropriate work schedules, posing challenges to pain management. A mismatch between the number of personnel, the number of patients, and the workload hinders nurses' ability to manage pain effectively. Nurses' experiences underscored the importance of having sufficient nursing and medical staff in the ward for effective pain management. High workloads and limited time were reported as obstacles to properly controlling patients’ pain. Many nurses expressed a desire to alleviate patients' pain but cited insufficient time and high workloads as significant obstacles in this regard.

*“Sometimes, shortages often prevent us from administering analgesics to patients promptly. When a patient reports pain, we may struggle to attend to them promptly as we must prioritize other patients, leading to increased suffering for the patient.” (P 14)*

#### Improper environment

Effective pain management extends beyond administering analgesics. Allowing patients adequate rest post-medication is essential for pain relief and medication efficacy. If the ward's physical layout hinders patient rest after receiving analgesics, it can impede pain relief significantly. Nurses in this study highlighted crowded wards and challenges in falling asleep after taking painkillers as barriers to optimal pain management.

*“In this ward, patients have received analgesics but are unable to rest due to the bustling environment. In my view, analgesics should be a last resort. Initially, we should focus on mentally preparing the patient, establishing a tranquil atmosphere, and then consider administering analgesics. However, the current practice is the reverse.” (P 16)*

### Personal barriers

Patients' individual characteristics, including prior painkiller usage and its side effects, past pain encounters, level of patient education, and physiological distinctions, can notably influence pain management. According to nurses' experiences, treatment resistance, fear of drug side effects, and patients' lack of knowledge and literacy were barriers to effective pain management.

#### Treatment resistance

Patients with similar types of cancer may experience varying levels of pain, each responding differently to analgesics, and exhibiting distinct sensitivities to the adverse effects of these drugs. Prolonged opioid use may lead to tolerance development, requiring dose escalation to sustain pain relief levels. Study participants stressed the variability of pain experiences among individuals, emphasizing healthcare professionals' consideration of individual tolerance levels and responses to analgesics, which can be influenced by physiological differences or prior drug use.

*“Each patient's pain is unique to their condition, requiring different dosages, types, and frequencies of analgesics, a fact that is often overlooked.” (P20)*

“*Sometimes, patients’ fear of using narcotics and the fear of dependence, coupled with societal stigma surrounding these drugs, lead patients to resist their usage.”(P5)*

*“Certain patients in this area perceive the illness as a matter of divine will, believing treatment should solely involve seeking divine intervention from God. Consequently, resistance to taking narcotic drugs poses a barrier to pain management.” (P4)*

#### Fear of drug side effects

Opioid use has been limited due to baseless worries, including the fear of dependency and potential side effects like drowsiness, constipation, urinary retention, dry mouth, along with more severe complications such as orthostatic hypotension, hepatotoxicity, and cardiotoxicity linked to prolonged analgesic use.

*“One of the challenges in pain management is the side effects associated with certain drugs; for instance, pethidine may induce seizures in patients after a few days, making it unsuitable for long-term use. Methadone, which can trigger apnea, is sometimes used as an alternative, further adding to patients’ concerns.” (P3)*

#### Patients' lack of knowledge

The majority of participants perceived health literacy as crucial in pain management. Low health literacy hinders effective communication between patients and healthcare providers, as well as understanding treatment instructions and the rationale behind prescriptions. Nurses highlighted that managing pain in patients with limited knowledge was notably more challenging than in well-informed patients, representing a major obstacle to optimal pain management.

*“Some patients have limited knowledge and struggle with communication and education, which poses a significant barrier to pain management.” (P2)*

## Discussion

The study aimed to identify barriers to pain management in cancer patients from the perspective of nurses in Iran. The study revealed several barriers, including the marginalization of complementary medicine, medical malpractice, inadequate organizational infrastructure, and personal barriers. Nurses pointed out factors such as lack of knowledge about complementary medicine, neglect of pain management, medical malpractice, treatment resistance, fear of drug side effects, and patients' lack of knowledge as reasons behind ineffective pain management. Numerous studies have also highlighted barriers to pain management, including lack of knowledge, poor communication, fear of complications, work pressure, and time constraints (23).

The study found insufficient personnel for non-pharmacological pain interventions in cancer patients due to healthcare providers' lack of knowledge and non-acceptance of these methods, as well as the common pharmaceutical culture in Iran. Doctors and nurses primarily rely on pain relief drugs for patients, disregarding non-pharmacological interventions. Other studies have highlighted the accessibility of opioids as the primary pain relief solution (24), inefficiency of patients’ companions, and reluctance to non-pharmacological measures (25). Additionally, obstacles to pain management include poor knowledge of non-pharmacological methods, lack of trust in their effectiveness (26, 27), negative attitudes among student nurses and nurses (28–30), physicians' insufficient training in complementary medicine, and inadequate scientific evidence supporting these methods (31). Some researchers have demonstrated that healthcare workers possess a high level of awareness of non-pharmacological pain management and complementary medicine, which contrasts with the current study results (32, 33). Positive experiences with complementary medicine have even led to its preference to chemical medicine (34). Despite the global importance of complementary medicine's role in pain relief and the presence of palliative care hospitals worldwide, healthcare workers in traditional Iranian society show little interest in these methods. This is attributed to the structure of healthcare centers, power hierarchies, and the limited role of nurses in patient-related decisions. This kind of research can help identify obstacles inhibiting the use of these methods and contribute to finding solutions.

The healthcare team's shortcomings, such as neglecting pain management and problems during medical visits, can pose important barriers to proper pain management. Some studies have highlighted obstacles such as physicians not prescribing painkillers, advising patients to tolerate pain without medication, and overlooking pain reports (16, 26). Nurses have reported instances of physicians refusing to prescribe pain medication, poor pain assessment, and reluctance to prescribe narcotics for cancer pain. Some healthcare providers believe that pain is inevitable in cancer, leading to a diminished sensitivity toward patients' pain relief (35). Poor knowledge, insufficient experience, and common misconceptions among healthcare providers regarding cancer pain pose significant challenges in pain management (36). Unlike the present study, a previous study indicated that Chinese physicians prioritize treating cancer pain as much as treating the cancer itself (37). Enhancing healthcare workers' knowledge and beliefs, starting from their education and continuing through training, can improve patient pain management (36).

Our results revealed that organizational limitations and background factors such as lack of personal and inappropriate environment can inhibit the use of non-pharmacological pain relief methods in cancer patients. While some researchers support these results, they suggest that the timely presence of nurses at the patient's bedside to manage pain can compensate for the lack of manpower, address time constraints, and reduce the workload of nurses (38). Other studies have reported challenges such as unbalanced employee workloads, inappropriate workplace, lack of time and heavy workload, insufficient staff, and lack of equipment, all of which hinder pain management in cancer patients (25, 39).

Our findings indicate that personal barriers such as treatment resistance, fear of drug side effects, and lack of knowledge of patients are significant barriers to effective pain management. These results are consistent with findings from other studies that have also highlighted fear of side effects such as cardiac arrest, addiction, constipation, nausea, vomiting, and fatigue due to a lack of understanding about drug side effects (40, 41, 42). Patients' negative attitudes towards the painkillers' efficacy, concerns about addiction, and fear of side effects have been reported as barriers to using pain drugs (26, 43). Workshops have been proven effective in increasing nurses' awareness and promoting proper pain management (40).

Our qualitative interviews with nurses at this institution offer valuable insights into the pain management processes. Nurses highlighted a system-related barrier: delays in pain interventions despite thorough pain assessments. These delays may stem from clinical providers underestimating pain severity, viewing pain as intrinsic to the disease, or due to inadequate organizational structures like insufficient staff for effective pain management. Overcoming these barriers will require substantial efforts, including comprehensive education at all levels to change culture and to improve workflow efficiency. Intervention should target reducing overall pain scores, minimizing pain-related errors, increasing patient satisfaction, and shortening hospital stays. Patients using opioids for pain management may face provider concerns about abuse and addiction, particularly due to opioid tolerance and high-dose requirements. These challenges underscore the need for multifaceted quality improvement initiatives to enhance patient pain management.

## Limitations and strengths

Our study's strengths lie in conducting in-depth interviews, having a single interviewer, and engaging the entire research team in data analysis. However, a limitation is the exclusive focus on nurses' perspectives, with potential insights from doctors or patients' families remaining untapped. Future research could explore physicians' or family patients’ perspectives on cancer pain management. Additionally, this study's limitation is its single-institution setting in Iran, limiting generalizability beyond this specific cultural and religious context.

## Conclusion

These findings provide valuable insights into the barriers impacting pain management for cancer patients. The success of pain management in practice depends on these influential factors in patient care. The present study revealed numerous challenges in pain management for cancer patients, such as the marginalization of complementary medicine, medical malpractice, inadequate organizational infrastructure, and personal barriers. Nursing policymakers can utilize these results to address obstacles such as staff shortage, by organizing educational workshops on complementary medicine and new pain management techniques to enhance healthcare workers’ understanding. Promoting humanistic care values and nursing professionalism can significantly improve the quality of nursing care, including pain management.

## Data Availability

The original contributions presented in the study are included in the article/Supplementary Material, further inquiries can be directed to the corresponding author.
